# Realising high aspect ratio 10 nm feature size in laser materials processing in air at 800 nm wavelength in the far-field by creating a high purity longitudinal light field at focus

**DOI:** 10.1038/s41377-022-00962-x

**Published:** 2022-12-02

**Authors:** Zhaoqing Li, Olivier Allegre, Lin Li

**Affiliations:** Department of Mechanical, Aerospace and Civil Engineering, The University of Manchester, Manchester, M13 9PL UK

**Keywords:** Optical physics, Laser material processing

## Abstract

In semiconductor and data storage device manufacturing, it is desirable to produce feature sizes less than 30 nm with a high depth-to-width aspect ratio on the target material rapidly at a low cost. However, optical diffraction limits the smallest focused laser beam diameter to around half of the laser wavelength (*λ*/2). The existing approach to achieving nanoscale fabrication is mainly based on costly extreme ultraviolet (EUV) technology operating within the diffraction limit. In this paper, a new method is shown to achieve materials processing resolution down to 10 nm (*λ*/80) at an infrared laser wavelength of around 800 nm in the far-field, in air, well beyond the optical diffraction limit. A high-quality longitudinal field with a purity of 94.7% is generated to realise this super-resolution. Both experiments and theoretical modelling have been carried out to verify and understand the findings. The ablation craters induced on polished silicon, copper, and sapphire are compared for different types of light fields. Holes of 10–30 nm in diameter are produced on sapphire with a depth-to-width aspect ratio of over 16 and a zero taper with a single pulse at 100–120 nJ pulse energy. Such high aspect ratio sub-50 nm holes produced with single pulse laser irradiation are rarely seen in laser processing, indicating a new material removal mechanism with the longitudinal field. The working distance (lens to target) is around 170 µm, thus the material processing is in the far field. Tapered nano-holes can also be produced by adjusting the lens to the target distance.

## Abstract (web summary)

Holes of 10–30 nm in diameter are produced on sapphire with a depth to width aspect ratio of over 16 and a zero taper with a single pulse longitudinal field.

## Graphical abstract

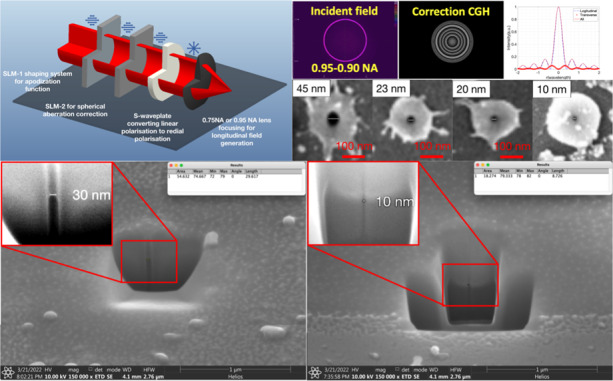

## Introduction

Towards the end of the 19th century, Abbe and Rayleigh formulated the principles of optical diffraction that limit the resolution of optical instruments in the far field where the distance between the target and the optical element is far greater than the optical wavelength^1^. In microscopy and astronomy, optical diffraction restricts imaging resolution thus reducing the ability to resolve small features and faraway objects. Similarly, in photolithography and laser processing, optical diffraction limits the ability to focus a laser beam to a spot much smaller than the laser wavelength^2^. Diffraction effects can be divided into Fresnel and Fraunhofer types. Fresnel diffraction affects optical interactions in the near-field (where the target to optics distance is at or smaller than the light wavelength) and Fraunhofer diffraction affects far-field interactions. In the far-field, the orientation of the electric field of light is typically transverse to the beam axis. At every point, the wavelet propagation paths are approximately parallel and meet each other to produce the Fraunhofer diffraction patterns^3^. In an inward-going field (e.g. a focused laser beam), the increase of the amplitude comes to a diffraction-limited axial point and stops when the spherical wavefront diameter reduces to *k*·*λ*/NA, where *k* is near unity and depends on the optical setup, *λ* is the wavelength, NA = *n*·sin *θ* is the numerical aperture, *n* is the refractive index of the medium between the target material and the objective lens (in air, *n* = 1.0003), *θ* is the maximal angular aperture convergence semi-angle with respect to the NA of the objective lens^4^. Several definitions for the diffraction limit are listed in Table 1, from which it can be seen that in the far-field, the minimum focal spot diameter is about half of the light wavelength in air or vacuum.

**Table 1:** Definitions of the diffraction limit of a light beam at focus

Abbe’s microscope diffraction limit	$\frac{{0.5\lambda }}{{{\mathrm {NA}}}}$
Rayleigh resolution criterion	$\frac{{0.61\lambda }}{{{\mathrm {NA}}}}$
Sparrow criterion	$\frac{{0.475\lambda }}{{{\mathrm {NA}}}}$
Houston criterion	$\frac{{0.515\lambda }}{{{\mathrm {NA}}}}$

The high demand for the manufacturing of functional micro- and nano-structures and devices in the last decades^5–7^ leads to a growing trend toward higher resolution. To increase the resolution in laser-based materials processing, one can either reduce the wavelength or use a higher NA lens to focus the beam, as shown in Table 1. A number of lithography techniques use very short wavelength beams^8^ to produce highly repeatable nanoscale features. Such techniques include electron beam lithography (EBL)^9^, focused ion beam (FIB) lithography^10^, X-ray lithography^11^, and plasmonic nanolithography^12^. However, all these techniques incur high costs and some of them require a vacuum environment, which complicates their operations. Photolithography is the main method for the precision manufacturing of semiconductors and optical devices. Patterns first produced on a photoresist are then transferred to the substrate using a combination of optical and chemical processes^13^. As a far-field super-resolution manufacturing technology, extreme ultraviolet (EUV) light is mainly applied in integrated circuit fabrication. By utilising a CO_2_ laser Xe or Sn plasma system, a 13.5 nm wavelength beam is generated for nanolithography. The drawbacks of the UV or EUV beam lithography include high cost, complex system structures and requiring a specific work environment (e.g., an ASML lithography system weighing around 180 tons and having over 100,000 components). Due to the strong absorption of UV and EUV light in air, ionisation of gases and potential damages to the optical components, EUV photolithography needs to be carried out in a high purity N_2_ gas chamber or a vacuum environment.

Another approach to achieving nano-scale resolutions in the optical processing of materials is using near-field methods. A number of these methods have been recently demonstrated, including microsphere lens^14^, nanosphere lithography^15^, scanning near-field optical lithography^16^, and scanning probe lithography (SPL)^17^. However, the throughput of near-field nanofabrication is relatively low due to the exponential decay of evanescent waves^18^. In addition, the working distance between the optical element and the target material is on the order of optical wavelengths, too small for many practical industrial applications and it could lead to damage to the optical system by process debris or collision.

Among the far-field methods, laser interference lithography^19^, metalens^20,21^, metamaterial super-lens^22^, femtosecond laser combined with stimulated emission and depletion (STED) approach^23^, and laser-induced periodic surface structures (LIPSS)^24^ have been demonstrated. A femtosecond laser beam has the advantage of multi-photon absorption, which helps realise sub-diffraction limit manufacturing^25^. During femtosecond laser direct writing, to realise the nanofabrication in the far-field, the laser fluence is often set low enough to ensure only the tip of the Gaussian beam is above the ablation or phase change threshold of the material. Features smaller than the optical diffraction limit can be generated. Normally, the pulse energy of femtosecond laser nanofabrication is 0.1–100 µJ and the laser optical power density is over 1 TW/cm^2^, although some heat-affected zones may still exist due to the melting threshold being lower than the ablation threshold and the laser-generated plasma lasting many nanoseconds^26^. During femtosecond laser processing, the generation of nano-features is attributed to the interaction between the beam, laser-induced plasma, and the material^27^. For linear exposure in femtosecond laser direct writing, materials respond to light excitation to the first order; while for two- and multi-photon absorption (nonlinear optical effect), two and higher orders are the main responses. The square light intensity distribution is spatially narrower than that of the linear one. The light–matter interaction volume can thus be reduced in multi-photon absorption so that the fabrication resolution is further improved^23^. Orthogonally polarised double femtosecond laser beams were applied to realise 12 nm features on a semiconductor surface based on the incubation effect^19^. However, such a feature cannot be generated without creating several features larger than 20 nm around, thus limiting its practical applications. For direct femtosecond laser ablation, it is still difficult to achieve a feature size at or below 10 nm due to Fraunhofer diffraction^28,29^.

Laser fields with a polarisation orientation parallel to their propagating axis are referred to as longitudinal fields. In principle, such longitudinal fields are incompatible with Maxwell’s equations. However, it has been shown theoretically that focusing radially polarised beams with a high numerical aperture (NA) lens can induce a longitudinal field component in the focal region^30^. By focusing an annular amplitude collimated laser beam with radial polarisation, opposite direction plane waves propagate towards the same focal point and cause constructive interference in the optical axis, leading to a symmetrical focal spot where all-the electric fields are oriented in the longitudinal direction, parallel to the optical axis. Early experimental applications of the longitudinal field include electron acceleration^31^, particle trapping^32^, Raman spectroscopy^33^, and three-dimensional photon crystal fabrication^34^. An attractive characteristic of the longitudinal field is the ability to break the diffraction limit^30,35^. The longitudinal field material processing can be a method potentially to break the Fraunhofer diffraction in the optical far-field. There have been several published reports to confirm the existence of longitudinal fields directly or indirectly. For instance, the first experimental demonstration of longitudinal field measurement was conducted through a knife-edge method using a 0.9–0.825 NA microscope objective lens^36^. The longitudinal field at the focal plane can be verified through the measurement of laser beam intensity distribution. A longitudinal field was also visualised through the generation of second harmonic (SH) patterns in a z-cut lithium niobate (LiNbO_3_) crystal using a 0.65 NA objective lens^37^. In the same publication, another method to detect the longitudinal field was using a qualitative measurement system to map the birefringence. An anisotropic structure with a slow axis perpendicular to the propagation direction of the laser beam confirms the existence of a longitudinal field^37^. However, the longitudinal field cannot be observed directly using these methods. The first material ablation using the longitudinal electric field of the laser beam was demonstrated on borosilicate Corning 0211 glass, by focusing an approximate radial polarisation laser beam through a 0.75–0.9 NA objective lens. The ablation crater with a diameter of around 100 nm was observed^38^.

Until now, although a number of investigations have been conducted to demonstrate the possibility to break the optical diffraction limit by using the longitudinal field^30,36–43^, to the best of our knowledge, high purity longitudinal fields have not been created for high-resolution materials processing, even though they are essential to achieving high processing resolutions. On the one hand, the spherical aberration of a high NA lens focusing is difficult to overcome by using standard optical lenses. In addition, the apodization function requires a high-quality annular collimated beam with a very small width and a uniform distribution, which is also difficult to realise by only blocking the central area of the collimated beam. If the focused laser beam is a mixture of longitudinal and transverse fields, the processing resolution is reduced.

In this paper, we experimentally demonstrate a longitudinal femtosecond laser field (i.e., parallel to the optical axis) with an unprecedented high purity (94.7%) and its interactions with polished copper, and sapphire. A new optical setup is developed using an 800 nm wavelength femtosecond laser source and a pair of spatial light modulators (SLMs) in a double 4-f optical arrangement to tailor the laser light fields to realise high-quality and uniform beam shaping for an apodization function (i.e. to generate a collimated narrow ring beam from a Gaussian beam) and to correct the spherical aberration of the 0.95 NA objective lens. The experiments involve focusing the beam using an aplanatic 0.75 NA lens to confirm the presence of the longitudinal fields, and the use of a 0.95 NA lens to understand the characteristics of the focused longitudinal fields. To understand the effect of the longitudinal field on laser materials processing, a number of polarisation states, beam intensity distribution and wavefront ablation profiles are compared. Theoretical modelling and discussion of the longitudinal field are given. Material processing with a resolution (10 nm, i.e., *λ*/80) well beyond the far-field diffraction limit is demonstrated on polished sapphire in air.

## Results

**Fig. 1:**
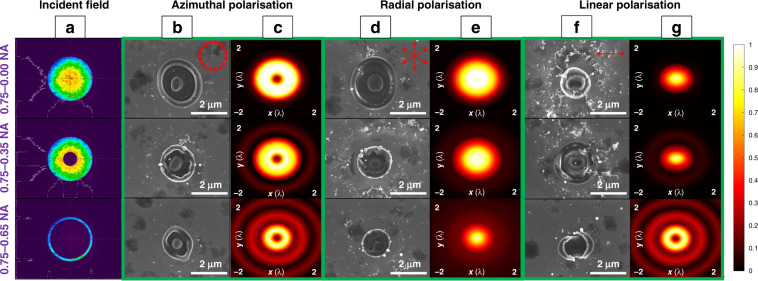
Laser intensity profiles and material processing effects with a 0.75 NA objective lens. **a** Laser beam intensity profile before the objective lens. The apodization function was set to be 0.75–0.00 NA (full aperture illumination, at the top), 0.75–0.35 NA (thick annular illumination, middle), and 0.75–0.65 NA (thin annular illumination, bottom). **b**, **d**, **f** Single pulse ablation on polished silicon with azimuthal, radial, and linear polarisation of laser beam under apodization function. **c**, **e**, **g** Corresponding modelled results of the beam intensity cross-section of the focused beam with azimuthal, radial, and linear polarisations under apodization function. The red symbols in **b**, **d** and **f** indicate the directions of E field with different polarisations

In the first set of experiments, the longitudinal field was confirmed by using a 0.75 NA lens with the apodization function. We focused the laser beam with linear, radial, and azimuthal polarisations with a 0.75 NA aplanatic objective lens. The beam shaping system induced the apodization function using the first SLM (noted SLM-1 henceforth), and the second SLM (noted SLM-2) was used as a mirror. By placing a linear polariser after a spatially micro-structured S-waveplate in two orthogonal directions and measuring the maximum and minimum average powers, the polarisation purity was determined at the position above the objective lens, which was 91.8%. Before laser materials processing, the collimated laser beam profile was measured using a SPIRICON beam profiler at the position of the objective lens aperture. Based on the measurement of the real aperture diameter of the objective lens whilst keeping the maximum NA and shaping the laser beam into 0.75–0.00 NA, 0.75–0.35 NA, and 0.75–0.65 NA for the objective lens apodization function, the measured intensity distribution of the collimated laser beam is shown in Fig. 1a. Laser materials processing was conducted under ambient condition. As shown in Fig. 1b, d, f, keeping the pulse energy at 43 nJ for all the polarisation and apodization functions, single pulse, the effect of laser ablation on polished silicon with different polarisation states under different apodization functions were compared. The lens to target distance was 380 µm. The focused light fields were also modelled theoretically and the intensity cross-section in the focal plane is shown in Fig. 1c, e, g. The model predicted that for the azimuthal polarisation, purely transverse annular fields were produced in the focal plane, as shown in Fig. 1c. A thinner annular beam created a smaller focal spot and more concentric rings, but the annular shape in the central zone was unchanged. The modelling result is consistent with the ablation result as shown in Fig. 1b. The pilar structure generated in the central zone was unchanged. The concentric rings visible in the model (Fig. 1c bottom) were not imprinted on the surface due to the low pulse energy. In Fig. 1e, for the radial polarisation under the thin annular beam illumination (bottom, the model indicated a purity of the longitudinal field of 74.1%), the focal spot became comparatively smaller and sharper. In the ablation result shown at the top of Fig. 1d, an unabated central pilar structure was visible. This indicated the longitudinal field presence in the centre was below the ablation threshold, i.e. not as strong as in the modelling result. This can be explained by the air/silicon interface effect, which makes the absorption efficiency of the longitudinal field lower than that of the transverse field due to the refractive index mismatch^37,40^. However, when the incident annular beam became thinner (Fig. 1a bottom), a flattop-like focal field was created (Fig. 1d bottom), which was induced by the combination of a transverse annular field and the central longitudinal field, leading to a near complete removal of the central pillar structure. The ablation of the central structure could only be achieved by the longitudinal field, the only non-zero component in the centre. In Fig. 1g bottom, for the linearly polarised laser beam, the thinner annular beam made the focal spot smaller and high-order concentric rings were generated. In Fig. 1f bottom, it can be seen that a cleaner ablation result was obtained using the thinner annular beam. High-order concentric rings were not generated under such low pulse energy. The results confirmed that under radial polarisation illumination with the apodization function set to a thin annular beam with 0.75–0.65 NA, the purity of the longitudinal field can be improved, even though the numerical aperture of the objective was as low as 0.75 NA. The longitudinal field was confirmed through single pulse ablation with this relatively low NA lens with the help of the apodization function. It was noticed that further reducing the pulse energy for processing on silicon, the flattop-like ablation pattern generated from the radial polarisation became an annular shape again, which is similar to the pattern generated by the azimuthal polarisation. It can also be explained by the low absorption efficiency resulting from the air/silicon interface effect of the longitudinal field from air to silicon^37,40^. In this investigation, silicon may not be the best material for isolating the longitudinal field. However, it was found that the combination and relative amplitude of the longitudinal and the transverse field had a significant impact on the ablation patterns.

**Fig. 2:**
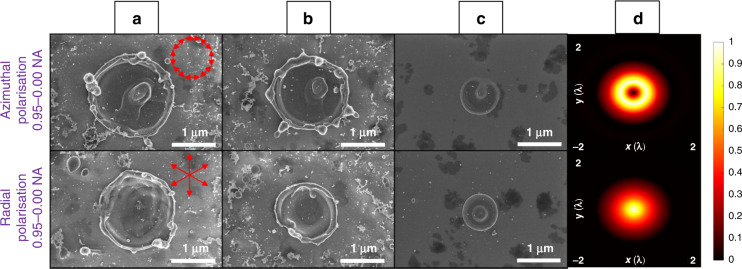
A comparison of azimuthal and racial polarisation effect on single pulse laser ablation on silicon at different pulse energy. Full aperture Gaussian beam single pulse ablation on polished silicon with azimuthal and radial polarisation under the pulse energy of **a** 210 nJ, **b** 120 nJ, and **c** 30 nJ, with a 0.95 NA objective lens. **d** The relative modelling of the beam intensity cross-section of the azimuthal and radial polarisations

In the second set of experiments, the longitudinal field was generated by using a 0.95 NA lens with spherical aberration correction, to characterise its interactions with silicon and copper. A tailored Fresnel wavefront was induced with SLM-2 to minimise spherical aberration of the 0.95 NA objective. The single pulse ablation of full aperture Gaussian beam with 0.95–0.00 NA focused on polished silicon is shown in Fig. 2a–c. The lens to target distance was 230 µm. It can be noticed that under different pulse energy, the azimuthal polarisation always created an annular shape in the focal plane. This is due to the field being zero in the centre. The radial polarisation beam created a flattop shape in the focal plane (similar in shape to Fig. 1d bottom with thin annular illumination and 0.75 NA lens focusing), even though no apodization function was applied here. This is due to the NA being significantly higher in this case. By dividing the peak intensity of the longitudinal field by the sum of the maximum intensity of the longitudinal and the transverse fields in the model, the purity of the longitudinal field was calculated to be 73.4% (comparable to the value of 74.1% obtained in Fig. 1d bottom with thin annular illumination and 0.75 NA lens focusing). The ablation craters produced with radial polarisation (i.e. a shallow bowl shape where the peak of material removal is at the centre) can only be explained by the presence of a longitudinal field in the centre, which merges with the annular transverse fields, i.e. only the longitudinal fields exist in the centre. This can be seen for example with the 30 nJ pulse energy radial polarisation ablation, which produced a relatively small hole in the middle of the footprint (Fig. 2c bottom). The ablation result is consistent with the numerical simulation as shown in Fig. 2d, which is also similar to the modelling result shown in Fig. 1, except that the high-order concentric rings were not generated under the full aperture beam illumination. According to our numerical simulation, the longitudinal field is the only non-zero field in the centre when focusing the radially polarised beam.

The longitudinal-to-total field ratio was calculated using the same method as above for the fields generated by radial polarisation beams focused by the 0.75 NA lens and the 0.95 NA lens with different inner NAs (referring to the numerical aperture of the blocked inner region of the laser beam on the objective lens). These figures referred to as longitudinal field purity (the longitudinal-to-total field ratio) henceforth, are shown in Table 2.

**Table 2:** Longitudinal-to-total field ratio, generated by radial polarisation beam focused by the 0.75 NA lens and the 0.95 NA lens with different inner NAs

	0.00 NA	0.10 NA	0.20 NA	0.30 NA	0.40 NA	0.50 NA	0.65 NA	0.80 NA	0.90 NA
0.75NA	54.3%	54.6%	55.7%	58.1%	61.8%	66.4%	74.1%	–	–
0.95NA	73.4%	73.5%	74.1%	75.6%	77.7%	80.6%	85.7%	91.2%	94.7%

Note that inner NA is 0.00 for all full aperture beams

**Fig. 3:**
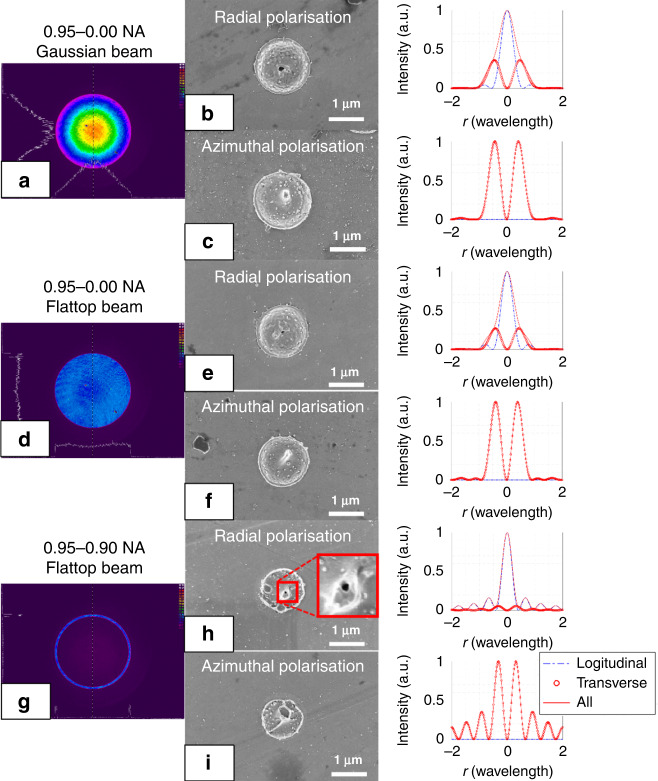
Comparison of effect of beam profile and polarisation state on laser ablation on copper. **a**, **d**, **g** Collimated laser beam intensity cross-section shaped into 0.95–0.00 NA Gaussian beam, 0.95–0.00 NA flattop beam, and 0.95–0.90 NA annular beam and detected by using a beam profiler. **b**, **c**, **e**, **f**, **h**, **i** Effect of single pulse ablation on polished copper and the result of relative focal spot modelling under radial and azimuthal polarisations

In addition to the spherical aberration correction and the application of apodization function, laser beam three-dimensional intensity distribution shaping based on the SLM-1 was carried out next. As shown in Fig. 3a, d, g, the collimated laser beam intensity distribution was set to be a full aperture Gaussian beam, a full aperture flattop beam, and a 0.95–0.90 NA annular beam and verified by using a beam profiler. Based on the spherical aberration correction and three-dimensional apodization function, laser beams with radial and azimuthal polarisation were applied for single pulse ablation with 210 nJ pulse energy on polished copper. As shown in Fig. 3b, under full aperture, radially polarised, Gaussian beam illumination (the purity of longitudinal field was 73.4%), a tiny hole with a diameter of around 110 nm was generated in the centre of the ablation pattern, while in Fig. 3c, the azimuthally polarised beam generated a pilar structure in the centre. The corresponding modelling results confirmed the intensity distribution at the focal position and the blue curve indicates the longitudinal field. In Fig. 3e, under full aperture flattop beam illumination (the purity of the longitudinal field was 78.7%), a tiny hole generated by using radial polarisation was reduced to around 80 nm, which is consistent with the enhancement of the longitudinal field in the modelling result. In Fig. 3h, i, the annular beam with 0.95–0.90 NA was applied for both radial (a purity of longitudinal field 94.7%) and azimuthal polarisation laser beams. The tiny hole in the centre of radial polarisation ablation was reduced to around 40 nm, while the azimuthal polarisation beam still generated an annular pattern.

**Fig. 4:**
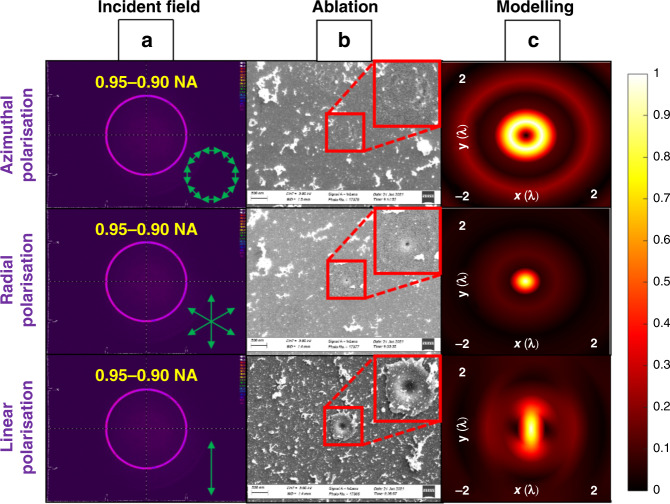
Comparison of effect of annular beam with different polarisation states on laser ablation on sapphire. **a** The intensity distribution of 0.95–0.90 NA annular Gaussian beam, **b** focused laser ablation with single pulses on polished sapphire (insets show ×2 magnified views, with increased contrast), **c** the normalised focal intensity distribution (modelling of the beam intensity cross-section) with azimuthal, radial, and linear polarisation (top to bottom respectively)

In the third set of experiments, the longitudinal field was enhanced using an apodization function for sapphire ablation, and the process characteristics were analysed in the same way as before. By using the beam apodization function based on SLM-1 to generate a thin annular collimated beam, spherical aberration can be reduced within a certain range. Figure 4a shows the beam profiler detection of collimated annular beams (0.95–0.90 NA, using SLM-1 to induce the apodization function. Since the polarisation singularity for radial and azimuthal polarisations can only be observed in the centre of the beam, which was zero in amplitude for an annular beam, for both three polarisations, the beam intensity distributions were the same. The polarisation states are marked in green). Figure 4b shows the focused laser beam single pulse ablation on a polished sapphire sheet with 260 nJ pulse energy. The polarisation states from top to bottom are azimuthal, radial, and linear polarisation, respectively. It can be observed that for single pulse ablation, the azimuthal polarisation created a very shallow annular crater, barely visible in Fig. 4b (top) due to the low fluence (the modelling result is shown in Fig. 4c top). The comparatively low fluence here is due to the annular intensity distribution that has a larger ablation area compared to the other polarisation fields. It is consistent with the property of the pure transverse component of the azimuthal polarisation. The annular linear polarisation beam single pulse ablation created a shallow wide bowl shape, with a diameter of around 180 nm, as shown in Fig. 4b bottom (the corresponding modelling result is shown in Fig. 4c bottom). The radial polarisation (with a longitudinal field purity of 94.7%) generated a tiny hole with a diameter of around 30 nm on the sapphire sheet, as shown in Fig. 4b middle with the corresponding modelling shown in Fig. 4c middle. The generation of the tiny hole confirms the advantage of the longitudinal field, which has a sharper intensity distribution or better resolution compared with the transverse field generated by azimuthal polarisation and linear polarisation. It is worth mentioning that even though the absorption efficiency of the longitudinal field is lower than that of the transverse field, by using the apodization function, most of the laser beam in the centre area of the Gaussian was removed so that there is little energy to contribute to the transverse field generation, which is the key factor for generating a high purity longitudinal field for the sub-diffraction limit manufacturing.

**Fig. 5:**
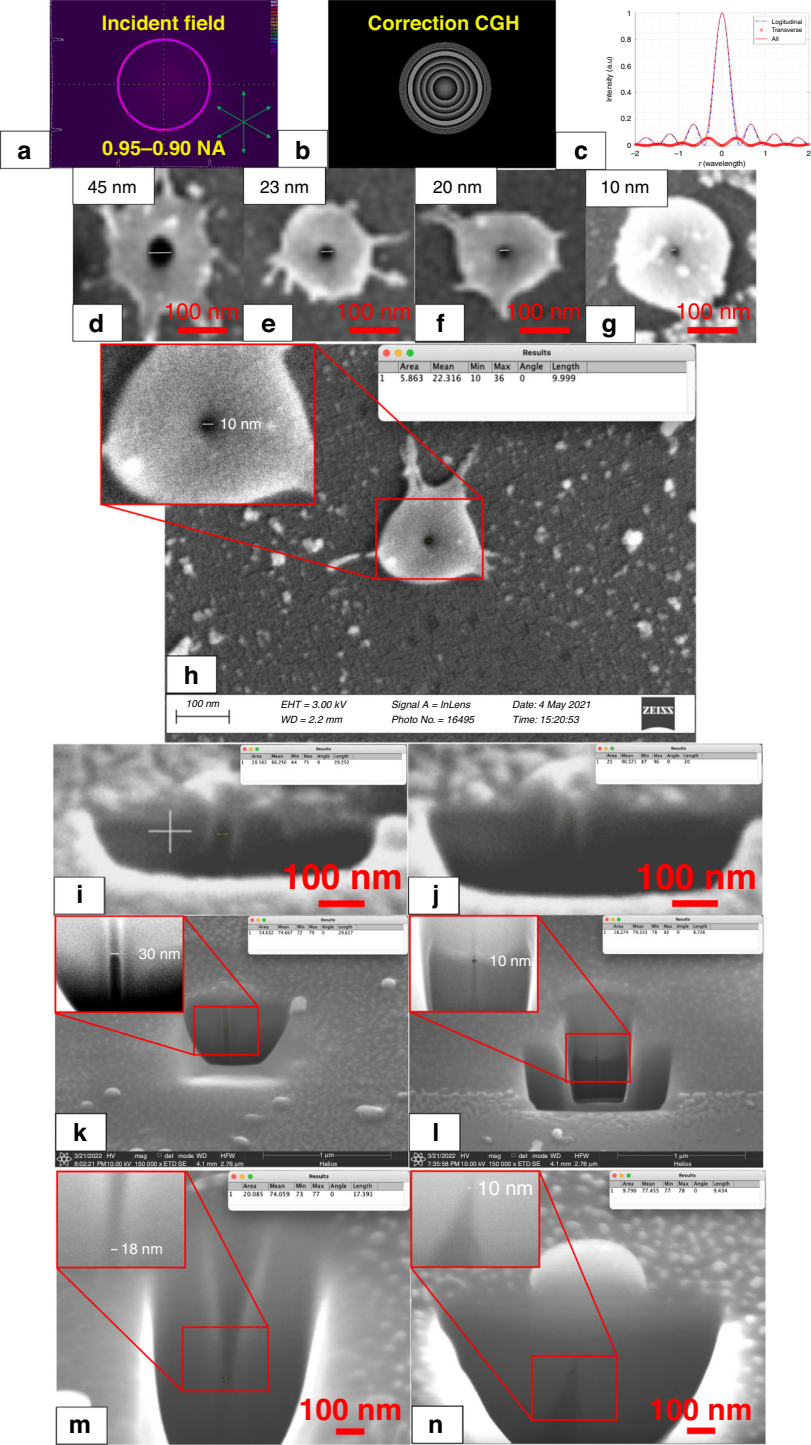
Effect of optimised longitudinal laser beam hole drilling on sapphire realising 10-30 nm hole size with high depth to width aspect ratio. **a** Intensity distribution of the radial polarisation annular beam based on apodization function measured before objective lens by using a beam profiler, **b** CGH for spherical aberration correction, **c** light field modelling for focusing radial polarisation with 0.95–0.90 NA objective lens, **d**–**h** effect of single pulse ablation based on laser beam with radial polarisation on polished sapphire with gradually reduced pulse energy, **i** and **j** the cross-section of holes with diameters of 30 and 20 nm, **k** the cross-section of a hole with no taper with a diameter of 30 nm and a depth of 500 nm, **l** the cross section showing a diameter of 8.7 nm extending to a depth of over 700 nm, **m** the cross-section of a hole with positive taper with a diameter down to 15.1 nm at the bottom area of taper, **n** the cross-section of a negative taper with a diameter down to 9.4 nm at the top area of taper

In the final set of experiments, the longitudinal field was further enhanced. In order to obtain a high-quality and high-purity longitudinal field, both the apodization function induced with SLM-1 and the spherical aberration correction through SLM-2 were employed. The collimated laser beam intensity distribution measured before the objective lens by using a beam profiler and the computer-generated hologram applied on the SLM-2 are shown Fig. 5a, b, respectively. 0.95–0.90 NA thin annular beams were used here to maximise the longitudinal field. A 94.7% purity of the longitudinal field can be obtained from the relative peak intensity of the longitudinal and transverse field components shown in Fig. 5c. Here, only radial polarisation was used, focusing the beam to generate a high-purity longitudinal field. Gradually reducing the pulse energy of the laser beam from 160 nJ to around 140, 120, and 100 nJ, the reproducible (more than 10 times repeated) feature size of the single pulse ablation on the polished sapphire could be reduced to hole diameters from 45 to 10 nm (as shown in Fig. 5d–h, in Fig. 5h the inset show ×2 magnified view, with increased contrast). The hole diameter and crosssection width measurements were made by using focused ion beam (FIB) cross-sectioning on an SEM machine and “ImageJ” feature measurement software, where the “Length” value in the “Results” inset represents the width of the feature in nanometres. The diameters were measured at the top of the holes directly after laser drilling, and for the cross-section after the FIB sectioning. The lens to target distance was 170 µm (due to the spherical aberration correction, the working distance was reduced from 230 µm), thus the material processing was still in the far field. As shown in Fig. 5g, h, holes with a diameter as small as 10 nm could be created, which is much smaller than the previously published results based on strong nonlinear laser materials interaction processes^25^. As can be seen in Fig. 5d–h, a ~200 nm diameter re-solidified molten material rim was also induced, and this facilitated locating the holes on the surface using SEM. It is expected this redeposited rim or spatters (all above the target material surface as confirmed by the cross-sectional views) can be removed by further optimising the parameters, as demonstrated by the result with radial polarisation in Fig. 4b (middle). To verify the cross-section characteristics of these tiny holes, FIB sectioning was conducted. The results are shown respectively in Fig. 5i–n. Figure 5i shows a hole diameter of 30 nm and a depth 90 nm with the deposited spatter shown above the workpiece surface. Figure 5j shows a diameter of 20 nm and a depth of 90 nm and parallel hole walls. Figure 5k shows a diameter of 30 nm and a depth of 500 nm (a depth-to-diameter aspect ratio of 16.6). Figure 5l shows a cross-section of 10 nm in width and over 700 nm in depth. The surface crater size was around 30 nm. The reduced internal width here compared to the surface crater size is due to the FIB cutting slightly offset from the centre of the hole. Here, the zero tapers and the greater depth of the hole can be confirmed. Figure 5m shows a positive taper with a minimum diameter of 15.1 nm at the bottom. To make this feature, the lens-to-target distance was increased. Figure 5n shows a negative taper with a minimum diameter of 9.4 nm at the top. To make this feature, the lens-to-target distance was reduced. In Fig. 5k–n, the inset show ×1.5 magnified view, with increased contrast. The results in Fig. 5k, l confirm that a high depth-to-width aspect ratio (more than 16) was realised. They also confirm that the surrounding materials observed on the top surface around the holes as shown in Fig. 5d–h were spatter redeposited on the surface, which could be removed or prevented by optimising laser processing parameters or the application of an anti-spatter spray. In Fig. 5n, the area above the taper top tip was invisible due to the resolution limit of the SEM. However, since the top tip of the negatively tapered hole was ultra-small (down to 9.4 nm), and the spatter was redeposited on the top surface of the material, there should be an ultra-small hole there to allow the material to escape from the lower part of the hole, which is consistent with the ultra-small feature as shown in Fig. 5g, h. The extremely small feature size and very high depth-to-width aspect ratio with parallel hole walls observed in this study could indicate that the material removal mechanism induced with the longitudinal field is fundamentally different from those induced with a transverse linear polarisation. This phenomenon is rarely seen in laser materials processing at this scale with a single pulse.

## Discussion

Comparing the ablation characteristics observed with linear and radial polarisations leads to interesting findings. For linear polarisation ablation with 260 nJ pulse energy, the single pulse ablation generated pattern was a shallow wide bowl, as shown in Fig. 4b bottom. Further reducing the pulse energy in linear polarisation, the ablation feature was not detectable. On the other hand, with radial polarisation inducing the longitudinal fields, reducing the pulse energy within the same range leads to a very gradual reduction in the ablation crater diameters as shown in Fig. 4b middle, and Fig. 5d–h. This indicates distinctly different ablation thresholds for these two polarisations pointing toward different ablation mechanisms.

Femtosecond surface ablation can produce crater sizes significantly below the diffraction limit through non-linear interactions, for example, multi-photon ionisation (MPI). With an 800 nm wavelength (~1.55 eV) laser beam, 7 photons are required to cross the ~10 eV bandgap of sapphire. This means laser ablation will be confined to the focal volume where intensity is above the threshold for 7-photon ionisation, which is a much smaller region than conventional optical diffraction limits such as those listed in Table 1. Thus, in theory, MPI could explain the crater diameters <60 nm seen in Fig. 5. Here it is interesting to compare the results obtained with a radial polarisation in Figs. 3h, 4b middle. Although these were both produced under similar laser irradiation parameters, the very different material characteristics of both materials would lead one to expect significantly different results. Sapphire is a wide bandgap material, and so non-linear material ablation mechanisms are expected to dominate, allowing one to produce ablation features below the optical diffraction limit. On the other hand, copper is a metal where free electrons will readily absorb laser pulse energy without non-linear effects, making it much more difficult to produce ablation craters significantly below the optical diffraction limit. Yet, under longitudinal field irradiation with pulse energy of 210–260 nJ, both these materials induced an ablation diameter of ~30–40 nm. Thus, conventional laser interactions are unlikely to explain the ablation craters in Fig. 5.

This leads us to believe the interaction is dominated by a mechanism specific to the longitudinal field which is not significantly affected by material properties such as bandgap. One such mechanism uses longitudinal laser fields at intensities >10^19^ W/cm^2^, irradiating a controlled gas target to accelerate electrons to relativistic speeds^44,45^. Although this is significantly different to irradiating the solid targets in our experiments, one may consider fast electron extraction from the surface, followed by Coulombic ion removal as a possible mechanism to explain our experimental results. The important parameter here is referred to as the normalised intensity parameter *a*_*z*_^2^ and is defined as^44,45^

1 $$a_z^2 \cong \eta _0I_0{{{\mathrm{exp(1)}}}}\left( {\frac{4}{{\pi ^2}}} \right)\left( {\frac{{\lambda _0}}{{w_0}}} \right)^2\left( {\frac{e}{{m_{\mathrm{e}}c\omega _{\mathrm{o}}}}} \right)^2$$

where *η*_0_ = 376.73 is the impedance of free space, *λ*_0_ = 8 × 10^−7^ m is the central wavelength, *e* = 1.6 × 10^−19^ C is the electron charge, *m*_e_ = 9.1 × 10^−31^ kg is the electron mass, *c* = 3 × 10^8^ m/s is the speed of light, *ω*_*o*_ = 2*πc*/*λ*_0_ = 2.356 × 10^15^ Hz is the angular frequency.

Based on the theory of electron acceleration^44^, when $a_z^2 \,>\, 1$, the amplitude of the normalised longitudinal field is in the relativistic regime where the relativistic energy is high enough to trap the electrons inside a half cycle of the laser field and accelerate it out of focus along the propagation axis. In our case, the pulse energy was 100 nJ, leading to $a_z^2\sim 10^{ - 3}$. This is significantly below the threshold and so direct relativistic electron extraction cannot be the dominant mechanism for ablation in our experiments.

The amplitude of the longitudinal electric field is defined as^44,45^

2 $$E_{\mathrm{L}} \cong \frac{{E_0 \cdot 2\surd (2 \cdot e)}}{{k_0 \cdot w_{\mathrm{o}}}}$$

where *E*_0_ is the peak amplitude of the transverse electric field and *k*_o_ = *ω*_o_/*c*.

Our experimental conditions lead to *E*_L_ ~ 10^11^ V/m. This is above the threshold for inducing Coulomb explosion, which is *E*_*th*_ ~ 5 × 10^10^ V/m for a sapphire. Our experimental results are unlikely to be induced from a standard Coulomb explosion with a transverse field^25^. For the standard Coulomb explosion, the transverse field of the ultrashort pulse laser beam transfers its energy to the electron system. The electrons are then ejected from the lattice by the high energy. In the experimental results demonstrated in this paper, the electrical field is in the vertical direction parallel to the laser beam axis. The laser beam with the longitudinal field could behave somewhat like a particle accelerator and make the electron (with a negative charge) and ion (positive charge) to be ejected more effectively than in a standard Coulombic explosion. In previously published papers, it has been demonstrated that an electron beam can be accelerated by using a longitudinal field^45^. The very deep holes produced in our experiments show the possibility of electron acceleration and charge polarisation in the holes for material removal.

**Fig. 6:**
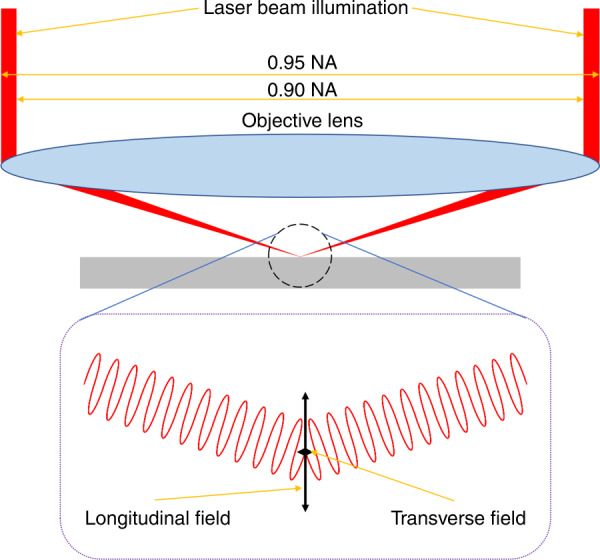
Schematic of the longitudinal field generation

The key driver to this interaction is the high purity of the longitudinal field. In our work, this was created using two spatial light modulators. The first SLM induced a high purity collimated thin annular beam and the second SLM corrected spherical aberrations (more details can be found in the “Materials and methods” section below). The laser beam with a radial polarisation was focused with a 0.95–0.90 NA objective lens onto the surface of the materials. Only in such a configuration, a high-purity longitudinal field could be generated. Our work demonstrates the highest purity (94.7%) longitudinal field so far, although as can be seen from the illustration in Fig. 6, 100% longitudinal field purity cannot be achieved. The results in Fig. 5 give confidence that the longitudinal field will allow laser processing with a resolution of 10 nm.

Figure 6 illustrates the high-purity longitudinal field generation at the focal plane. This is based on the experimental configuration using both the apodization function (to block laser beam in low NA and only allow high NA part pass through, which is essential for high purity longitudinal field generation, even though the pulse energy efficiency is very low) and the spherical aberration correction (to correct the laser beam focus in longitudinal orientation, to ensure the collimated annular beam is focused in the same plane under high NA).

## Materials and methods

### Materials

In this study, the longitudinal field was generated to induce ablation craters on polished silicon, copper, and sapphire.

### Analytical formulation of focused laser fields

Several methods can be used to describe a focused beam under high NA^46^. Richards–Wolf vectorial diffraction is generally used to model optical fields focused with a high NA lens^47^. Assuming an aplanatic lens is used to converge the incident collimated beam with a spherical wave into the focal sphere, a diffraction-limited axial point image can be generated.

For *x*-polarised linear polarisation focusing, the electrical focal field can be expressed as^48^

3 $$\mathop{E}\limits^{\rightharpoonup} \left( {\rho ,\varphi ,z} \right) = - i\frac{{2\pi f}}{\lambda }\left\{ {\left[ {I_0 + \cos \left( {2\varphi } \right)I_2} \right]\mathop{e}\limits^{\rightharpoonup} \!{_x} + \sin \left( {2\varphi } \right)I_2\mathop{e}\limits^{\rightharpoonup} \!{_y} - 2i\cos\varphi I_1\mathop{e}\limits^{\rightharpoonup} \!{_z}} \right\}$$

where $\mathop{e}\limits^{\rightharpoonup} \!{_x}$, $\mathop{e}\limits^{\rightharpoonup} \!{_y}$, $\mathop{e}\limits^{\rightharpoonup} \!{_z}$ are the unit vectors in the transverse directions *x* and *y* and longitudinal direction *z*, respectively, and the definition of three variables *I*_0_, *I*_1_, *I*_2_ is given by

4 $$I_0 = {\int}_{\theta _{{\mathrm{min}}}}^{\theta _{{\mathrm{max}}}} {\ell \left( \theta \right)P\left( \theta \right)\sin\theta (1 + \cos\theta )J_0(k\rho \sin\theta )e^{ikz\cos\theta }{\mathrm{d}}\theta }$$

5 $$I_1 = {\int}_{\theta _{{\mathrm{min}}}}^{\theta _{{\mathrm{max}}}} {\ell \left( \theta \right)P\left( \theta \right)\sin\theta \sin\theta J_1(k\rho \sin\theta )e^{ikz\cos\theta }{\mathrm{d}}\theta }$$

6 $$I_2 = {\int}_{\theta _{{\mathrm{min}}}}^{\theta _{{\mathrm{max}}}} {\ell \left( \theta \right)P\left( \theta \right)\sin\theta (1 - \cos\theta )J_2(k\rho \sin\theta )e^{ikz\cos\theta }{\mathrm{d}}\theta }$$

*J*_*n*_ (*kρ* sin *θ*) is the Bessel function of the first kind of order *n*

7 $$J_n\left( x \right) = \mathop {\sum}\limits_{m = 0}^\infty {\frac{{\left( { - 1} \right)^m}}{{m!\Gamma \left( {m + n + 1} \right)}}\left( {\frac{x}{2}} \right)^{2m + n}}$$

The electrical focal field of a cylindrical vector (CV) beam in cylindrical coordinates can be express as^43^

8 $$\mathop{E}\limits^{\rightharpoonup} \left( {\rho ,\varphi ,z} \right) = E_\rho \mathop{e}\limits^{\rightharpoonup} \!\!{_\rho} + E_\varphi \mathop{e}\limits^{\rightharpoonup} \!\!{_\varphi} + E_z\mathop{e}\limits^{\rightharpoonup} \!\!{_z}$$

where $\mathop{e}\limits^{\rightharpoonup} \!{_\rho}$, $\mathop{e}\limits^{\rightharpoonup} \!{_\varphi}$, $\mathop{e}\limits^{\rightharpoonup} \!{_z}$ are the unit vectors in radial, azimuthal, and longitudinal directions and *E*_*ρ*_, *E*_*φ*_, *E*_*z*_ are the amplitudes in radial, azimuthal, and longitudinal directions.

9 $$E_\rho \left( {\rho ,\varphi ,z} \right) = \frac{{2\pi f}}{\lambda }\cos\varphi _0{\int}_{\theta _{min}}^{\theta _{{\mathrm{max}}}} {\ell \left( \theta \right)P\left( \theta \right)\sin\theta \cos\theta J_1(k\rho \sin\theta )e^{ikz\cos\theta }{\mathrm{d}}\theta }$$

10 $$E_\varphi \left( {\rho ,\varphi ,z} \right) = \frac{{2\pi f}}{\lambda }\sin\varphi _0{\int}_{\theta _{{\mathrm{min}}}}^{\theta _{{\mathrm{max}}}} {\ell \left( \theta \right)P\left( \theta \right)\sin\theta J_1(k\rho \sin\theta )e^{ikz\cos\theta }{\mathrm{d}}\theta }$$

11 $$E_z\left( {\rho ,\varphi ,z} \right) = - i\frac{{2\pi f}}{\lambda }\cos\varphi _0{\int}_{\theta _{{\mathrm{min}}}}^{\theta _{{\mathrm{max}}}} {\ell \left( \theta \right)P\left( \theta \right)\sin\theta \sin\theta J_0(k\rho \sin\theta )e^{ikz\cos\theta }{\mathrm{d}}\theta }$$

where *f* is the focal length of the objective lens and *λ* is the wavelength, *φ*_0_ refers to the azimuthal angle of the incident electric field, for radial polarisation incident beam, *φ*_0_ = 0, for azimuthal polarisation incident beam, *φ*_0_ = *π*. *α*_max_ and *α*_min_ refer to the maximal and minimum angular aperture half-angle (convergence semi-angle) respect with NA of the objective lens, and refer to the prescribed pupil apodization function about the amplitude tailored field, which is a relative angular amplitude distribution of the collimated incident electric field pupil entrance, maintains cylindrical symmetry and may vary radially^49^.

12 $$\ell \left( \theta \right) = {{{\mathrm{exp}}}}\left[ { - \beta ^2\left( {\frac{{\sin\theta }}{{\sin\theta _{{\mathrm{max}}}}}} \right)^2} \right]$$

where *β* is a truncation parameter, as the ratio of the pupil radius *R* and the beam waist *w*.

13 $$\beta = \frac{R}{w} = 1$$

*P*(*θ*) is the pupil plane anodization factor, in this paper, the objective lens obeys the sine condition, and has

14 $$P\left( \theta \right) = \sqrt {\cos\theta }$$

*k* is the wavenumber

15 $$k = \frac{{2\pi }}{\lambda }$$

All components are independent of *φ*. It can be considered that all components have cylindrical symmetry. All length measurements are normalised to the unit of wavelength *λ*. The intra-focal contour plot of electric energy density can be created at both the focal plane and through focus by applying the squared electric field components

16 $$I = \left| E \right|^2$$

All modelling was conducted under Matlab 2020, and the output modelling results are shown together with ablation results in later sections.

### Experimental method

**Fig. 7:**
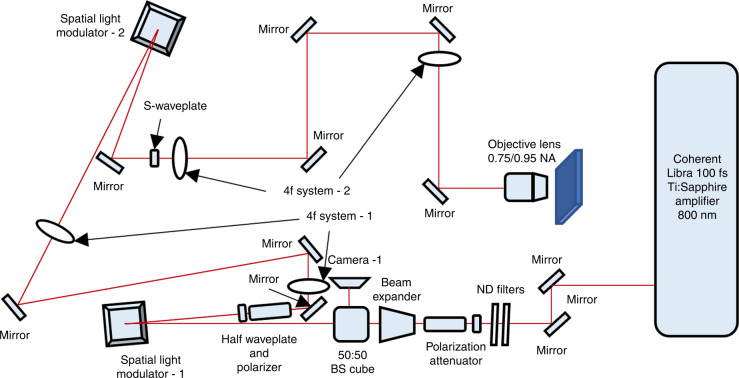
An illustration of the experimental setup

**Fig. 8:**
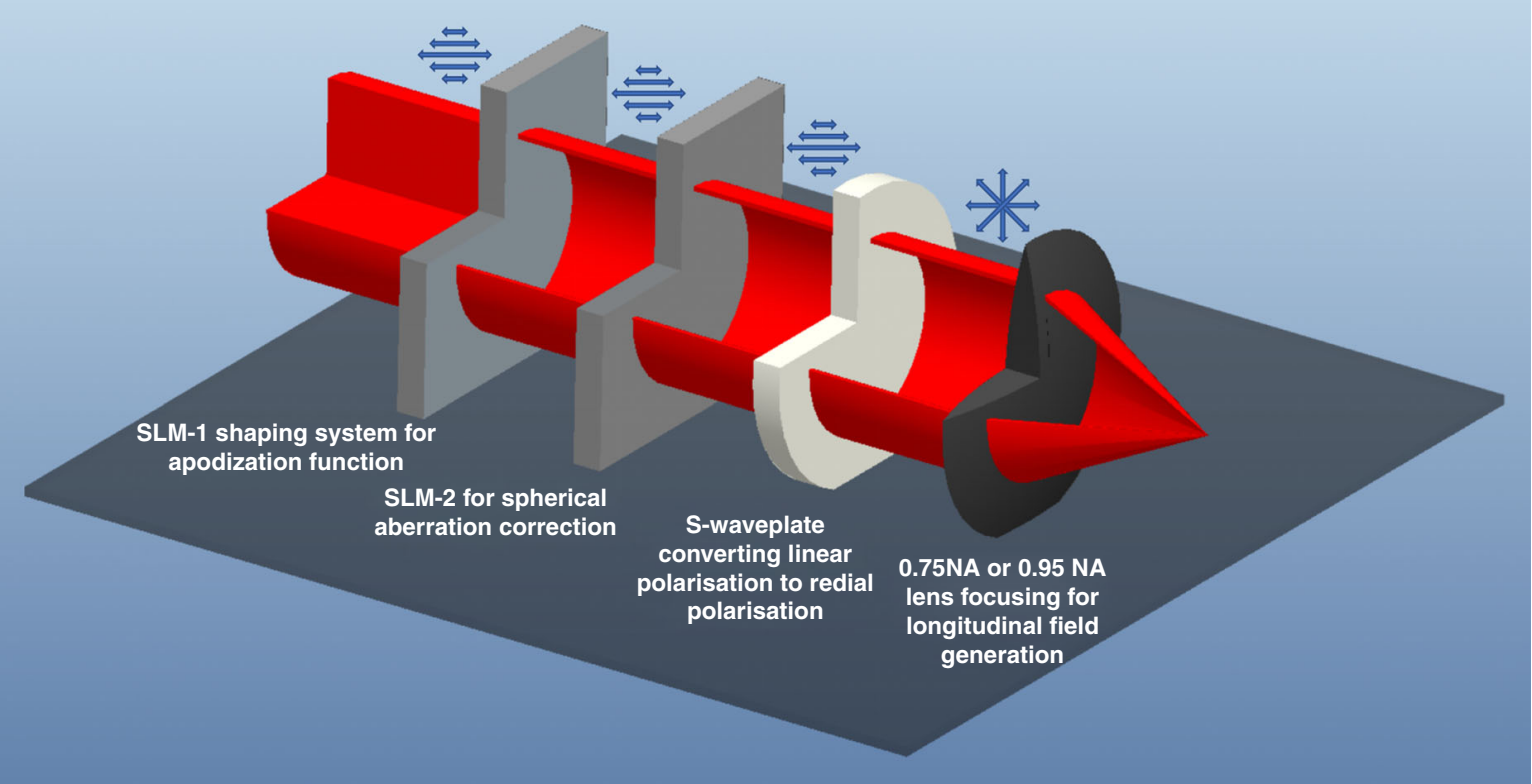
A 3D diagram showing the principle of longitudinal field generation

The experimental setup is shown in Fig. 7. The laser source was a Coherent Libra Ti: Sapphire (100 fs pulse duration, an 800 nm wavelength, at 1 kHz repetition rate). The pulse energy of the laser beam was controlled by using neutral density (ND) filters and a polarisation attenuator. After a 2× beam expander, the laser beam was illuminated onto a beam shaping system, which is a combination of a spatial light modulator (SLM-1, Hamamatsu X10468-02), a half waveplate and a polarizer. The beam shaping system in the beam path was for the generation of the apodization function (i.e., to generate a parallel annular beam) so that the direction of the inserted linear polarisation is at 45° to the optical axis of liquid crystals in SLM-1. To keep the quality of the light field, a 4-f lens system was applied to reconstruct the laser beam in SLM-1 to SLM-2 (Hamamatsu X10468-07). The second SLM was employed for the laser beam wavefront tailoring (i.e., spherical aberration correction), so that the inserted linear polarisation is in the direction parallel to the liquid crystal in SLM-2. Another 4-f system was used to reconstruct the laser beam in SLM-2 to the aperture of the objective lens. A fused silica S-waveplate (Altechna) was applied in the beam path to convert linear polarisation into radial polarisation or azimuthal polarisation. Finally, the laser beam was focused through an OLYMPUS 0.75 NA MPLan N ×50 objective lens or a Nikon 0.95 NA CFI Plan Apo Lambda 60XC objective lens. The second lens has a spherical aberration when the laser is focused on a surface without a cover glass (which can be corrected by using SLM-2). All samples were placed on an Aerotech high-resolution three-axis translation stage with a resolution of about 50 nm. To maintain the polarisation purity, Thorlabs low-group-delay-dispersion, thin-film-coated ultrafast mirrors were applied in all the beam paths. The principle of the longitudinal field generation is illustrated in Fig. 8. The original Gaussian beam with linear polarisation was shaped into an annular shape, followed by apodization correction, and radial polarisation, successively, and finally focused with a 0.95 NA objective lens.

### Spherical aberration correction

**Fig. 9:**
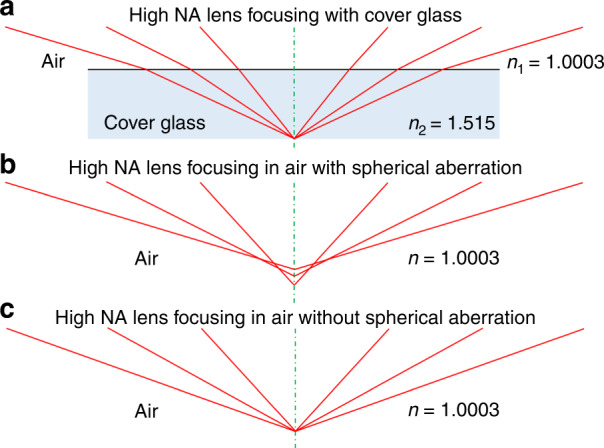
Illustration of role of spherical aberration effect and its correction in laser beam focusing. Comparison of high NA lens **a** focused with cover glass, **b** focused in the air with spherical aberration, and **c** focused in the air without spherical aberration

To focus the laser beam to a sub-wavelength scale, one of the main challenges is to create a perfect spherical cage. In this paper, the 0.95 NA objective lens applied was designed for microscope observation with a cover glass of 0.9 mm in thickness. Without the cover glass, the laser beam would be focused on a non-uniform range, instead of a single point. The schematic of the beam focusing is shown in Fig. 9.

**Fig. 10:**
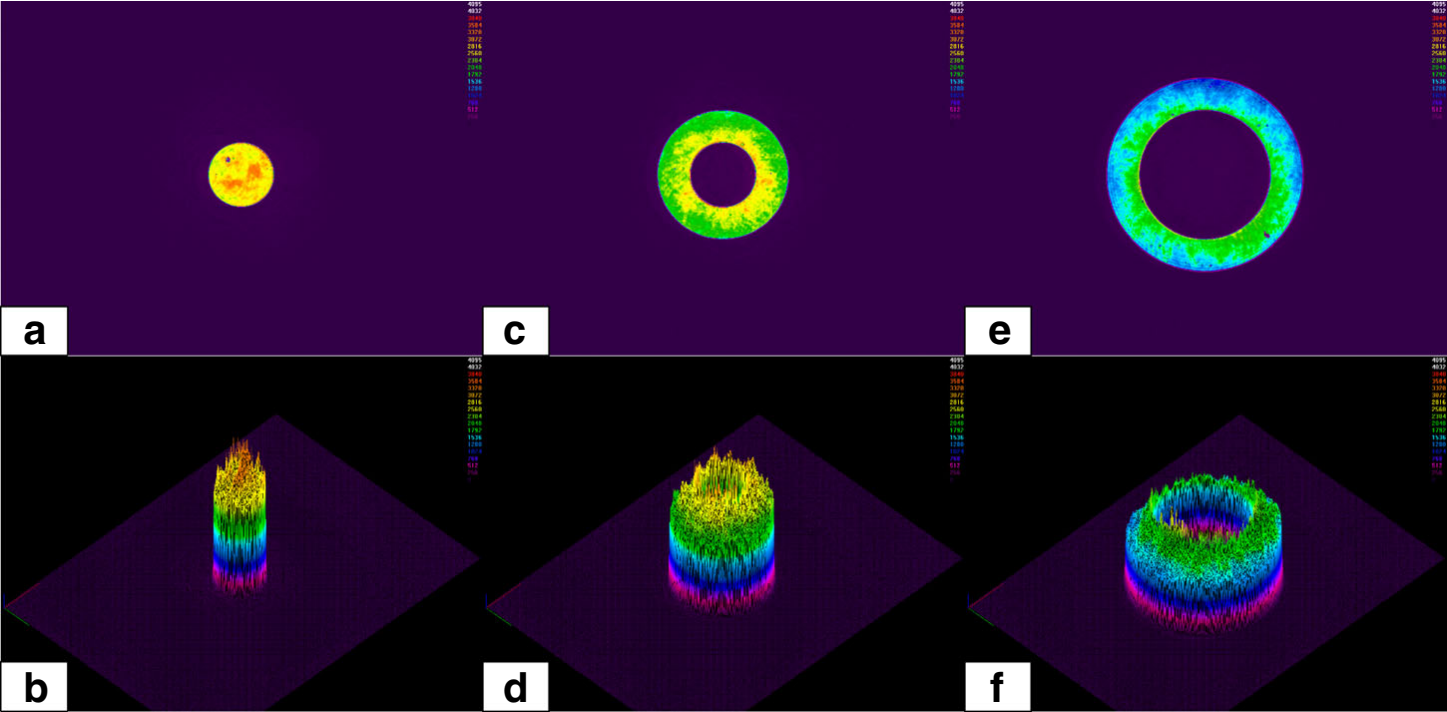
Beam shaping of the original laser beam, the NA were 0.30–0.00, 0.60–0.30, and 0.95–0.60 from left to right

To quantify and correct the spherical aberration, a novel method was employed based on the beam shaping technology and the radial direction processing strategy. First of all, we used apodization functions to shape the collimated laser beam to be different diameters, which refers to the different NAs of the 0.95 NA objective lens. As shown in Fig. 10, from left to right they were 0.30–0.00 NA, 0.60–0.30 NA, and 0.95–0.60 NA.

**Fig. 11:**
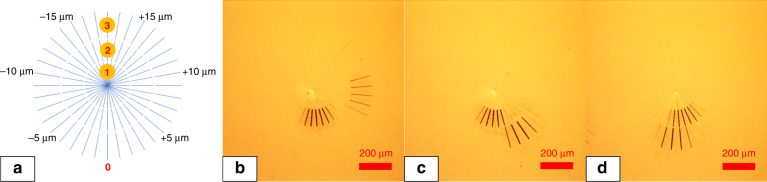
Laser beam processing strategy to quantify and correct the spherical aberration

We developed a processing strategy to visualise and quantify spherical aberration and experimentally verify its correct compensation. As shown in Fig. 11a, we used a beam scanning path that followed a radial orientation. Three concentric radial patterns were inscribed on a silicon surface, numbered 1, 2, and 3 in Fig. 11a, where the laser beam had been shaped with apodization functions of NA of 0.30–0.00, 0.60–0.30 and 0.95–0.60, respectively. This meant that the inner radial pattern 1 had been inscribed with the inner part of the beam (shown in Fig. 10a) and the outer radial pattern 3, with the outer regions of the beam shown in Fig. 10e. The incremental step between each neighbouring lines was 1 µm in the vertical (*z*) direction. When the top surface of the sample was out of the depth of focus, there would be no ablation occurring. In this way, one can easily define the focal plane by recognising the relative angle from zero degrees (marked in red in Fig. 11a). Thus, the distribution of focal planes of the 0.95 NA lens with spherical aberration can be located. For example, in Fig. 11b, it can be found that the laser beams with NA of 0.30–0.00, 0.60–0.30, and 0.95–0.60 were focused on different focal planes (the scanning speed was 1 mm/s, the pulse energy were measured to be 60, 92, and 110 nJ, respectively). The distance between the 0.30–0.00 NA focal plane and 0.95–0.60 NA focal plane was about 17 µm (the focal length of 0.95–0.60 NA was more than 17 µm longer than the focal length of 0.30–0.00 NA), which is due to the spherical aberration. By applying a tailored Fresnel lens wavefront correction CGH on SLM-2, spherical aberration correction can be realised. The equation to generate the wavefront correction CGH is^50^

17 $$\hat \emptyset _{{\mathrm {SA}}}\left( \rho \right) = \frac{{2\pi d_{{\mathrm {nom}}}}}{\lambda }\left( {\sqrt {n_1^2 - ({\mathrm {NA}}\rho )^2} - \sqrt {n_2^2 - ({\mathrm {NA}}\rho )^2} } \right)$$

where *d*_nom_ is the nominal focal depth, which is set to be 0.059 mm, the scale for cover glass was set to be 0.09 mm (actual depth), *λ* is the light wavelength, *ρ* is the normalised pupil radius. The refractive index of air and the cover glass are *n*_1_ = 1.0003, and *n*_2_ = 1.515, respectively.

Figure 11c, d shows the focal plane position gradually optimised based on the spherical aberration correction. In Fig. 11d, it can be found that the focal positions for both 0.30–0.00 NA, 0.60–0.30 NA, and 0.95–0.60 NA are in the same plane so that the spherical aberration is removed.

### Beam intensity distribution modulation

**Fig. 12:**
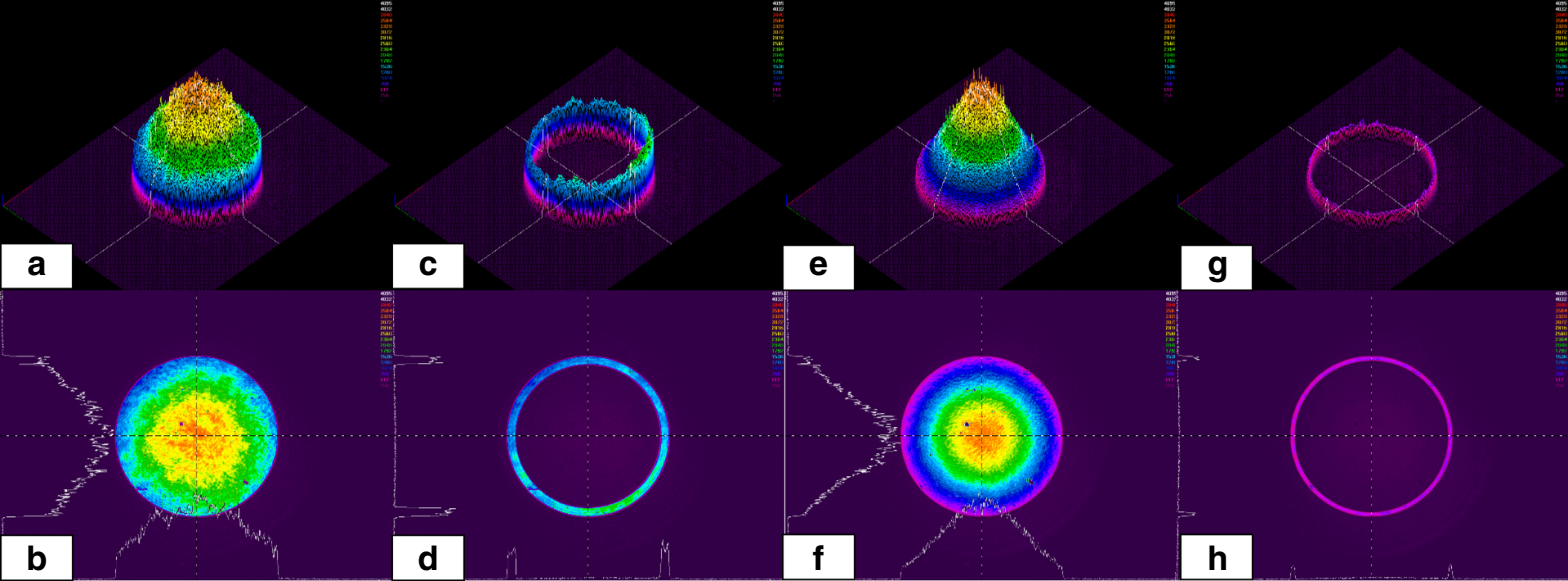
Experimental measurement result of laser beam profiles before and after beam shaping. Comparison of collimated **a**, **b** original full aperture Gaussian beam, **c**, **d** original annular Gaussian beam, and **e**, **f** shaped full aperture Gaussian beam, **g**, **h** shaped annular Gaussian beam

To obtain a high-quality and high-purity longitudinal field, not only the spherical aberration needs to be removed, but also the intensity distribution needs to be optimised. When the laser beam is reflected or transmitted through so many optical elements in the beam path, the beam mode has gradually deteriorated. Figure 12 shows the comparison of the collimated original Gaussian beam and the shaped Gaussian beam, detected before the laser beam is illuminated into the aperture of the objective lens by using a beam profiler. In Fig. 12a–d, it can be noticed that there are some beam mode distortions in the original Gaussian beam. Under the apodization function, the generated annular beam is ununiform. Due to the fact that the longitudinal field is generated from the interference of the laser beam in all the spherical directions, any non-uniformity of the annular beam will make the longitudinal field mixed with the transverse field in some orientations. As shown in Fig. 12e–h, the shaped beam is very close to the standard Gaussian beam intensity distribution. Through the apodization functioning, the uniformity of the annular beam is almost perfect, which is quite important for high-quality longitudinal field generation.
